# RACE AND ETHNICITY DISPARITIES IN SUBJECTIVE COGNITIVE DECLINE

**DOI:** 10.1093/geroni/igz038.3091

**Published:** 2019-11-08

**Authors:** Nia Reed, Christopher A Taylor, Benjamin Olivari, Karen Wooten, Lisa C McGuire

**Affiliations:** 1 Centers for Disease Control and Prevention, Atlanta, Georgia, United States; 2 CDC, Atlanta, Georgia, United States; 3 DB Consulting, Silver Springs, Maryland, United States; 4 Centers for Disease and Prevention, Alzheimer’s Disease and Healthy Aging Program, Atlanta, Georgia, United States

## Abstract

Alzheimer’s disease (AD) is the most common form of dementia. Subjective cognitive decline (SCD) is the self-reported experience of worsening or more frequent confusion or memory loss and it is one of the earliest noticeable symptoms of AD. Data from respondents aged 45 years and older to the Centers for Disease Control and Prevention’s Behavioral Risk Factor Surveillance System Cognitive Decline module were examined to identify race and ethnicity disparities in SCD. This module was administered by 49 participating states, District of Columbia, and Puerto Rico from 2015-2018. Data were analyzed using SAS statistical software and methods that accounted for survey design and weighted data. Prevalence of SCD by race/ethnicity with 95% confidence intervals (CI) was calculated. Among adults aged 45 years and older, one in nine (10.8%; CI=10.5-11.2) non-Hispanic white adults experienced SCD. In comparison, among adults aged 45 years and older, one in nine (11.2%; CI=9.8-12.7) Hispanic, one in eight (13.2%; CI=12.0-14.3) African American/black, and one in five (19.6%; CI=16.0-23.2) American Indian/Alaska Native (AI/AN) adults experienced SCD. These numbers are expected to increase significantly over time, especially for some minority groups. More specifically, Hispanics and African Americans are expected to constitute a large proportion of older adults in the coming decades. There are implications in how communities are reached with respect to awareness of cognitive decline (this includes AI/AN adults, as well). Race and ethnicity disparities in SCD may be influenced by differences in chronic diseases and other risk factors that are also disparate between communities.

